# Light quality induces a shift in coccosphere morphology in *Scyphosphaera apsteinii*

**DOI:** 10.1093/plankt/fbae032

**Published:** 2024-06-12

**Authors:** Nishant Chauhan, Samuel Barton, Stergios Zarkogiannis, Rosalind E M Rickaby

**Affiliations:** Department of Earth Sciences, University of Oxford, South Parks Road, OX1 3AN, UK; Department of Earth Sciences, University of Oxford, South Parks Road, OX1 3AN, UK; Department of Earth Sciences, University of Oxford, South Parks Road, OX1 3AN, UK; Department of Earth Sciences, University of Oxford, South Parks Road, OX1 3AN, UK

**Keywords:** calcification, coccolithophores, light

## Abstract

The coccolithophore *Schyphosphaera apsteinii* produces distinct coccolith morphotypes and offers a unique insight into coccolith calcification, as the number of lopadoliths per cell increases under low light intensities. This study employs *S. apsteinii* to investigate the acclimated impact of light intensity and wavelength on cell physiology and coccosphere morphology. Our findings reveal a marked increase in lopadolith production when grown under reduced light intensity, with lower growth rates, higher chlorophyll concentration and elevated net photosynthetic rates. Reduced blue-light also caused an increase in lopadolith numbers, elevated chlorophyll concentrations and net photosynthetic rates. Conversely, such responses are less pronounced under reduced red-light. Moreover, reduced blue- and red-light treatments exhibited enhanced growth rates compared to the light-replete control, despite a reduction in light intensity. Our findings suggest that changes in light quality cause a shift in the coccosphere morphology, affecting cell physiology and potentially aiding light harvesting in *S. apsteinii*.

## INTRODUCTION

Coccolithophores are a diverse group of marine microalgae that play a pivotal role in the global carbon cycle through their calcification and photosynthetic potential (Ridgwell *et al*., 2009). They create calcite plates (coccoliths) intracellularly, and post-exocytosis these form the cell’s outer shell (Young *et al*., 1999). Coccoliths are key to understanding biological control on calcification, which governs the chemistry and morphology of the calcite (Mackinder *et al*., 2010; Brownlee *et al*., 2021). However, the function of coccoliths is yet unknown (Raven and Crawfurd, 2012; Monteiro *et al*., 2016). Within coccolithophores, an intriguing but understudied order is the Zygodiscales. *Scyphosphaera apsteinii* provides a unique opportunity to investigate coccolith formation as it produces distinct coccolith morphotypes: barrel-shaped lopadoliths, plate-like muroliths and various intermediates in the diploid life-cycle phase (Drescher *et al*., 2012).

Few studies have investigated the mechanism of coccolith formation in this species. Drescher *et al*. (2012) first reported the inverse relationship between light intensity and the number of barrel-shaped lopadoliths in *S. apsteinii*, which indicates the potential function of coccoliths in light capture, as previously proposed (Sikes and Wilbur, 1982; Monteiro *et al*., 2016). Although the natural habitat depth of *S. apsteinii* is not well known, there is some evidence for its prevalence at depths of 60 m (e.g. Liu *et al*., 2020). The potential light-harnessing abilities of lopadoliths could prove useful at such depths which are beyond the penetration depth of longer wavelengths (e.g. red region of the visible spectrum) (Siesser, 1998; Holtrop *et al*., 2020). To investigate whether lopadoliths may enhance growth under varying light intensity and wavelength, we studied the physiology and coccolith morphology of *S. apsteinii* under varying light quality (intensity and wavelength).

## METHODS

*S. apsteinii* cultures were grown in Aquil synthetic seawater (Morel *et al*., 1979; Price *et al*., 1989) enriched with L1 nutrients (Guillard and Hargraves, 1993) under a 14:10 light:dark cycle at 17°C. Cultures were grown long-term under standard incubator light conditions (Fluorescent light: Panasonic FL40SS ENW/37, Japan; “natural colour”; 64.9 ± 13.4 μmol m^−2^ s^−1^), which was also used as “control.” Triplicate cultures were also grown in reduced light intensity (through neutral density filters; 13.8 ± 2.7 μmol m^−2^ s^−1^) transmitting the same wavelength spectrum as “control,” reduced blue-light (transmitting 505–700 nm; 51.2 ± 8.5 μmol m^−2^ s^−1^) and reduced red-light (transmitting 400–575 nm; 34 ± 6.0 μmol m^−2^ s^−1^) conditions (Fig. S2). Specific wavelength filters were obtained from Lee Filters and transmitted < 40% light for the inhibiting wavelengths (See Supplementary Information for details). Cultures were acclimated for ~ 2 months prior to making the measurements described below, all of which were obtained during exponential growth (Fig. S1). Growth was monitored using TECAN Spark® Microplate Reader and Beckman Z2 Coulter Counter. Chlorophyll *a* concentration was determined using the protocol by (Warren, 2008) and equations from (Ritchie, 2006). Scanning electron micrographs were taken on a Zeiss Sigma 300 FEG-SEM, and the number of lopadoliths per cell was subsequently counted (approx. 300 cells per treatment). Acute responses of net photosynthesis (NP) to white light conditions were quantified for all treatments using a clark-type oxygen electrode in combination with a Hansatech Instruments Oxylab system (using LED1/WHITE Hansatech lights). NP rates were measured across a gradient of light intensities from 0 to 1950 μmol m^−2^ s^−1^ to obtain a photosynthesis-irradiance (PI) curve and derive a value of maximal performance, *NP_max_*, which was quantified using the Eiler’s photoinhibition model (Eilers and Peeters, 1988). Further methodology and data used in this study are available in the Supplementary Material.

## RESULTS AND DISCUSSION

**Fig. 1:**
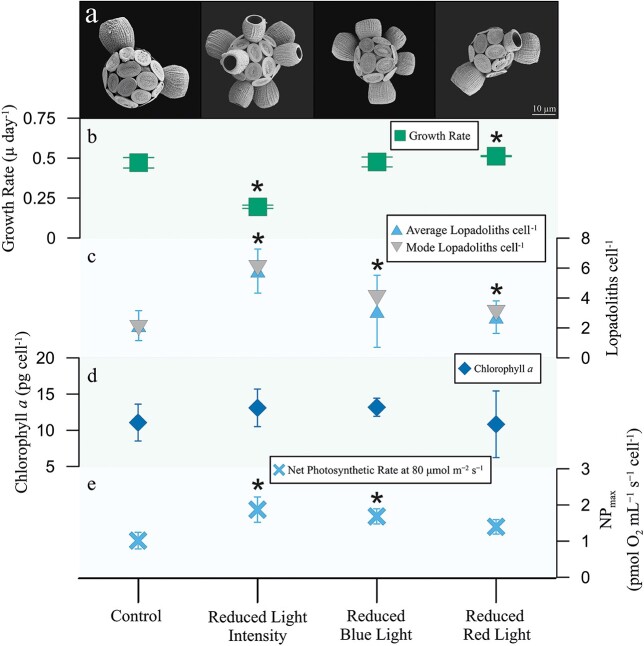
(a) Scanning electron micrographs for *S. apsteinii* under Control Light, Reduced Light Intensity, Reduced Blue Light and Reduced Red Light treatments (from left to right). Treatments included changes in light wavelength and intensity. (b) Growth rates (day^−1^) showing significant changes between Control and Reduced Light Intensity (*P* < 0.05), Control and Reduced Red Light (*P* < 0.05), Reduced Light Intensity and Reduced Red Light (*P* < 0.05) and Reduced Light Intensity and Reduced Blue Light (*P* < 0.05). (c) Average and mode of number of lopadoliths cell^−1^. Significant differences were observed between all treatments (*P* < 0.0007, for all pairwise comparisons). Number of lopadoliths was counted on at least 300 cells for each treatment. (d) Average chlorophyll *a* concentration (pg cell^−1^). Changes in concentration between treatments were statistically insignificant due to large errors and small replicate numbers (*n* = 9 per treatment) (e) Acute NP_max_ (pmol O_2_ cell^−1^ hr^−1^). Changes in NP_max_ were statistically significant between Control and Reduced Light Intensity (*P* = 0.03), and Control and Reduced Blue Light (*P* = 0.02). Full PI curves are provided in the Supplementary Information. Asterisks indicate statistical significance compared to control only; refer to Supplementary Information for comparison between treatments and further details on statistical tests. Error bars represent one standard deviation.

This study investigated the impact of light quality (absence of certain wavelengths and/or reduced light intensity) on physiological changes and changes in coccosphere morphology in *S. apsteinii*. We observed significant alteration in coccosphere morphology and growth rates of *S. apsteinii* in response to changing light quality (Fig. 1a and b). Growth rates significantly declined under reduced light intensity (14 μmol m^−2^ s^−1^) but showed a significant increase under reduced red light, despite the decrease in light intensity (transmitting 400–575 nm; 34 μmol m^−2^ s^−1^). Concurrently, number of lopadoliths increased under reduced light intensity, consistent with findings by Drescher *et al*. (2012) (Fig. 1a and c). Moreover, we observed an increase in lopadoliths cell^−1^ in *S. apsteinii* under reduced blue-light (transmitting 505–700 nm; 51 μmol m^−2^ s^−1^), despite small changes in the light intensity compared to the control, which exhibited fewer lopadoliths cell^−1^. A marginal increase in lopadolith numbers was observed in the reduced red-light treatment, relative to the control. The higher number of lopadoliths cell^−1^ under reduced blue and red light was partly attributed to the increase in lopadolith-murolith intermediates (Fig. S3). Additionally, the presence of a higher number of lopadolith-murolith intermediates under reduced blue and red light coincided with elevated growth rates (Fig. 1b and c; Table I), despite a decrease in light intensity and wavelength range. This may suggest that light quality influences the costs and benefits of lopadolith calcification in *S. apsteinii* (Raven, 1984) or that lopadoliths may influence light-harvesting capabilities of *S. apsteinii* under conditions of reduced light quality (Monteiro *et al*., 2016). For instance, intermediate-phase lopadoliths may form due to faster overturning of coccoliths when growth rates are faster in reduced blue- and red-light (Fig. 1). Moreover, the long, complete lopadoliths of the cells in reduced light intensity may be a consequence of slower growth rates, when a given coccolith spends longer time within the coccolith vesicle.

**Table I:** Table showing the key parameters utilized in this study

Treatment	Growth rate (day^−1^)	Light intensity (μmol m^−2^ s^−1^)	Avg. lopadoliths cell^−1^	Mode lopadoliths cell^−1^	Chlorophyll *a* (pg cell^−1^)	*NP_max_* (pmol O_2_ cell^−1^ hr^−1^)	Cell diameter (μm)
Control	0.25 ± 0.03	64.9 ± 13.4	2.16 ± 1	2	11.07 ± 2.55	1.02 ± 0.23	16.7 ± 0.5
Reduced light intensity	0.18 ± 0.01	13.8 ± 2.7	5.8 ± 1.5	6	13.09 ± 2.6	1.87 ± 0.35	17.8 ± 0.8
Reduced blue light	0.28 ± 0.03	51.2 ± 8.5	3.12 ± 2.4	4	13.18 ± 1.25	1.68 ± 0.21	16.5 ± 0.4
Reduced red light	0.33 ± 0.0	34 ± 6	2.72 ± 1.1	3	10.82 ± 4.61	1.39 ± 0.20	17.7 ± 0.6

 Changes in light quality induced notable physiological responses in *S. apsteinii*. An increase in chlorophyll *a* cell^−1^ was evident under changing light quality, especially with reduced light intensity, and reduced blue-light (Fig. 1d). This could signify a cellular adaptation aimed at enhancing light-harvesting capabilities, potentially explaining the observed higher growth rates under reduced blue light and red light (Mohsenpour *et al*., 2012). However, this cannot be confirmed as changes in chlorophyll *a* per cell were statistically insignificant, and there were no notable alterations observed in the absorption spectrum (Fig. S4). NP rates increased alongside chlorophyll concentrations. Notably, cells grown under reduced blue-light displayed higher acute NP rates than the control or red-light-limited cells across the full PI response (Fig. 1e; Table I; Table S1, S2; Fig. S5), indicating heightened photosynthetic potential due to blue light deprivation. No significant changes in cell size were found across all the treatments (Table I), and therefore, it can be inferred that the observed increase in per cell chlorophyll *a* and NP rates in both reduced light intensity and reduced blue-light treatments is not attributable to size, but perhaps to subcellular physiological adjustments such as increased chlorophyll *a* concentration. While red light is necessary given the double peak of chlorophyll absorbance (Fig. S4), the data suggest that blue light exerts a stronger influence over physiology and calcification.

In conclusion, this study highlights the impact of light quality (both intensity and wavelength) on physiology and coccosphere morphology of *S. apsteinii*. We propose the hypothesis that lopadoliths may play a role in light harvesting, particularly if *S. apsteinii* inhabits deeper photic zones where predominantly blue light penetrates (Liu *et al*., 2020). Moreover, we demonstrate that lopadolith production is more likely associated with blue wavelength limitation, as opposed to red. This presents novel insights into the dynamics of coccosphere morphology as a photoresponse and raises questions about the evolutionary origins of the two coccolith morphologies in *S. apsteinii.* Nevertheless, a more comprehensive investigation of the light capture mechanisms of lopadoliths, particularly under constant light intensity but with wavelength limitations, is imperative to fully elucidate these findings.

## Supplementary Material

Supplementary_Material_fbae032

## Data Availability

All data used in this study are available in the Supplementary Material or at: https://doi.org/10.5281/zenodo.10204082.
