# Supplementary material for: Light quality induces a shift in coccosphere morphology in Scyphosphaera apsteinii
Source: J Plankton Res. 2024 Jun 12;46(4):383–6. doi: 10.1093/plankt/fbae032 (PMC11290244; doi:10.1093/plankt/fbae032)
Supplement: Supplementary_Material_fbae032 [file supplementary_material_fbae032.docx]

**Supplementary Material**

for

**Title:** Light quality induces a shift in coccosphere morphology in *Scyphosphaera apsteinii*

**Authors:** Nishant Chauhan^1,*^, Samuel Barton^1^, Stergios Zarkogiannis^1*^, Rosalind E.M. Rickaby^1^

**Affiliations:** ^1^Department of Earth Sciences, University of Oxford, U.K.

***Corresponding Author:** [nishant.chauhan@earth.ox.ac.uk](mailto:nishant.chauhan@earth.ox.ac.uk) (N. Chauhan)

stergios.zarkogiannis@earth.ox.ac.uk (S. Zarkogiannis)

**Methodology:**

**Culture:**

Monocultures of *Scyphosphaera apsteinii* (RCC 1456) were obtained from Roscoff Culture Collection. Cell sizes were measured on a Beckman Z2 Coulter Counter. Briefly, Cultures were diluted 1:10 in 3.5% NaCl and decalcified using 2 – 4 drops of 0.01N HCl prior to measuring the cell sizes. Coccosphere sizes were not measured on the Coulter Counter as the presence of lopadoliths gave erroneous coccosphere diameter values.

**Growth rates:**

Growth rates were calculated using a linear fit on log transformed chlorophyll fluorescence data obtained from the TECAN plate reader. A minimum of 7 points from the exponential growth phase were used to obtain growth rates from triplicate cultures for each treatment. Chlorophyll fluorescence was used instead of cell counts to allow more accurate determination of growth as chlorophyll pigment concentration and fluorescence is reported to have negligible changes within a treatment once acclimated (Marra, 1978). Moreover, in vivo chlorophyll fluorescence has been used for measuring growth rates in the past (Andersen, 2005).

**Chlorophyll Quantification:**

50 mL of culture was centrifuged in light safe centrifuge tubes, and chlorophyll was extracted overnight using 100% ethanol at 4°C. The samples were centrifuged once again to separate the extracted chlorophyll from cell debris. Chlorophyll *a* absorbance was measured in triplicates for each biological replicate on a TECAN Spark® Multimode Microplate Reader at 667 nm and chlorophyll *c* was measured at 626 nm, with a bandwidth of 30 nm and the values were obtained using the following equation given by (Ritchie, 2006)

𝐶ℎ𝑙𝑜𝑟𝑜𝑝ℎ𝑦𝑙𝑙 𝑎 𝑐𝑒𝑙𝑙^-1^ = (−1.4014×A_𝐶ℎ𝑙𝑜𝑟𝑜𝑝ℎ𝑦𝑙𝑙 𝑐_) + (12.1551×A_𝐶ℎ𝑙𝑜𝑟𝑜𝑝ℎ𝑦𝑙𝑙 𝑎_) ×10^6^

where A_𝐶ℎ𝑙𝑜𝑟𝑜𝑝ℎ𝑦𝑙𝑙 𝑎_ and A_𝐶ℎ𝑙𝑜𝑟𝑜𝑝ℎ𝑦𝑙𝑙 𝑐_ represent their respective blank-corrected absorbance values. A constant path length correction was applied to the samples based on the path length determined from water (Warren, 2008).

**Experimental Data Acquisition:**

Growth was monitored regularly post inoculation to monitor acclimatisation (See figure below). Collection of physiological data commenced 56 days after initial inoculation and after 4 transfers. Culture transfer into fresh media always occurred in the mid exponential phase and below a cell density of 25,000 cells mL^-1^. Due to technical constraints, the PI curve was not measured at the same timepoint. The cultures were maintained for a further 71 days after which, the measurements for the PI curve were taken. Growth rates were observed during this time and did not change drastically.


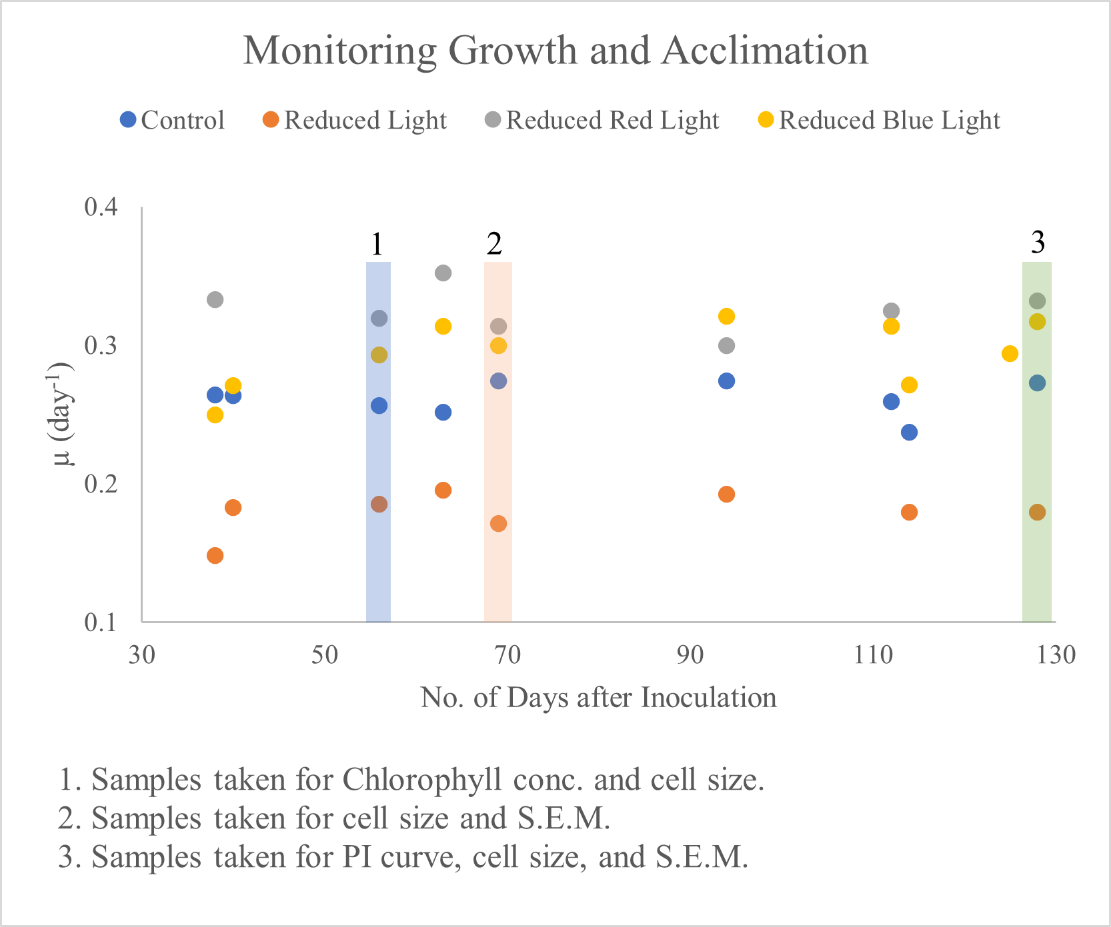


Figure S1: Long-term growth rates for Scyphosphaera apsteinii.

**Light Intensity and Wavelength:**

Filters for Reduced Blue Light and Reduced Red Light were bought from Lee filters (www.leefilters.com). Filter “101 Yellow” was used for blue light limitation, which allowed 0 % light between 400 – 475, 30 % at 495 nm, and 50 % light at 505nm and >80 % at 535 nm and above. Filter “117 Steel Blue” was used for red light limitation and allowed >80% light at light below 515 nm wavelength, and 20 – 30 % between wavelengths of 610 and 685 nm. Reduced light conditions were obtained by using standard neutral density filters. Light intensity was measured for each treatment using a digital light meter.


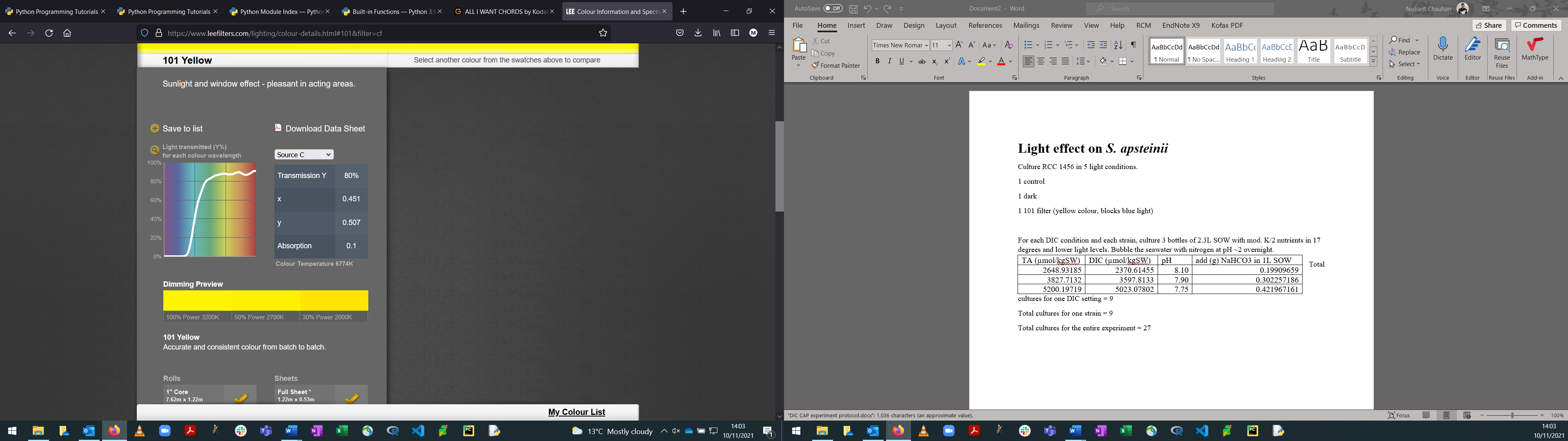

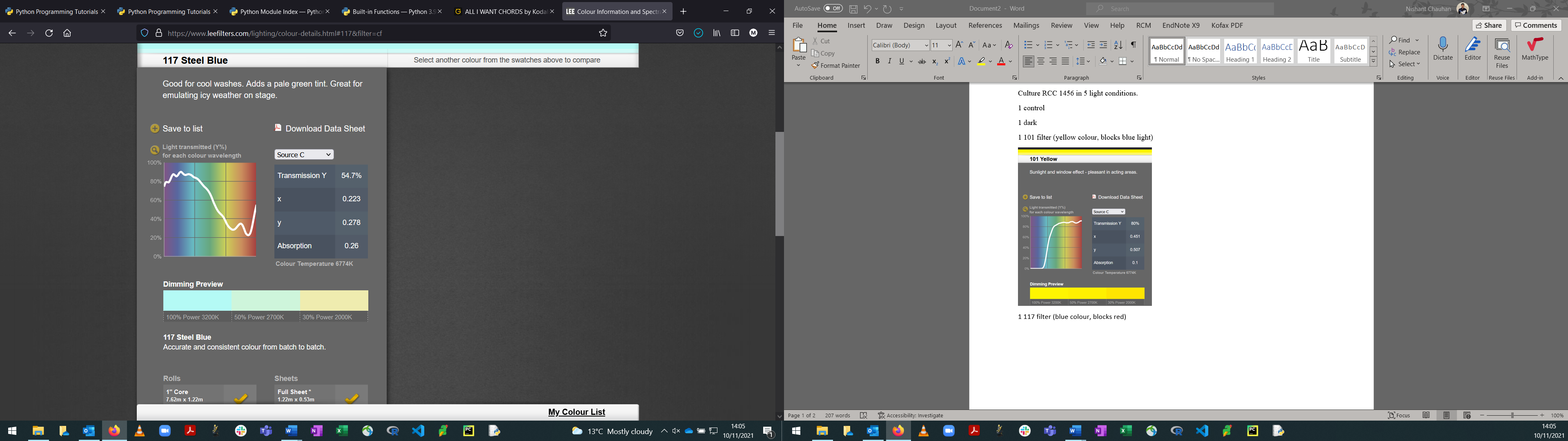


Figure S2: Light filters used for blue- and red-light-limitation (obtained from www.leefilters.com)*.*

**Counting the number of lopadoliths cell^-1^**

Lopadoliths visible in the SEM images were only counted from intact cells. At least 300 cells were analysed for each treatment from several replicate SEM images. 300 cells were chosen as the sample size to ensure statistical accuracy based on recommendations from prior literature (Kline, 1986; Bujang and Adnan, 2016). Since SEM images provided only a 2-dimensional view, all visible lopadoliths were counted. Lopadolith-murolith intermediates were counted as 0.5 unit per intermediate. Moreover, they were only counted if their height was at least ~50 % of the average mature lopadolith height, that is, an intermediate was only counted if it was ~6 µm or taller.

**Quantification of the Photosynthesis-Irradiance Curve:**

Measurements for the PI curve was made at the same temperature (17°C) as growth. A circulating water bath was used to regulate temperature. Cultures were centrifuged gently (at 500g for 3 minutes) to concentrate cells for the measurements. 1mL of this concentrated culture used. The cell density was obtained using a Coulter Counter. Samples were given a minimum of 10 minutes to pre-acclimate in the dark prior to the commencement of the measurement. PI measurements were made for 5 minutes at each light level above 80 μmol m^−2^ s^−1^. PI measurements at 0 (darkness) and at 80 μmol m^−2^ s^−1^ were made for 10 minutes. The background drift of a blank sample (filter sterilised blank seawater) was used to correct raw rate data before modelling the PI response. Eiler’s photoinhibition model was used to quantify the PI curve using nonlinear least squares regression:

$$NP \left( I \right)=\frac{{NP}_{max}I}{\frac{{NP}_{max}}{a{I_{opt}}^{2}}I^{2}+\left( 1-2\frac{{NP}_{max}}{{aI}_{opt}} \right)I + \frac{{NP}_{max}}{a}}$$

Here, *NP*(*I*) is the rate of net primary production at light intensity *I.* *I* was measured in μmol m^−2^ s^−1^. *NP_max_* is the maximum *NP* rate at optimal light intensity, *I_opt_*_._ *α* is the rate at which *NP* increases up to *NP_max_*.

**Statistical Analysis:**

Statistical tests such as interaction-two-way ANOVA, independent t-tests, pairwise comparison, Wilcoxon Rank Sum tests, Shapiro tests and Kruskal–Wallis *H* tests were conducted on the dataset based on the distribution of data (quantified graphically as well as using Shapiro test). All analyses were conducted in R.

**Results:**

**Statistical analysis:**

Statistical analysis on **growth rates** were done on rates obtained for each triplicate at Point 3 (Fig.S1). t-test on growth rates provided the following results:

| **treatment** | **t** | **df** | **p-value** |
| --- | --- | --- | --- |
| Control - Reduced Light | 3.8341 | 2.439 | **0.04469** |
| Control - Reduced Blue Light | 1.2247 | 4 | 0.2879 |
| Control - Reduced Red Light | -4.3818 | 2.439 | **0.03333** |
| Reduced Light - Reduced Red Light | 18.371 | 4 | **5.17x10^-05^** |
| Reduced Light - Reduced Blue Light | 5.4772 | 2.439 | **0.02012** |
| Reduced Blue Light - Reduced Red Light | -2.7386 | 2.439 | 0.0898 |

Welch Two Sample t-tests also suggested that the **number of lopadoliths per cell** changed significantly between each treatment (n = 300 per treatment):

| **Treatment** | **t** | **Df** | **P value** |
| --- | --- | --- | --- |
| Control - Reduced Light | 30.741 | 343.74 | **< 2.2x10^-16^** |
| Control - Reduced Blue Light | 8.0234 | 470.18 | **8.272x10^-15^** |
| Control - Reduced Red Light | 6.9144 | 260.57 | **3.615x10^-11^** |
| Reduced Light - Reduced Red Light | -28.331 | 520.32 | **< 2.2x10^-16^** |
| Reduced Light - Reduced Blue Light | -16.675 | 598.37 | **< 2.2x10^-16^** |
| Reduced Blue Light - Reduced Red Light | -3.4282 | 514.65 | **0.0006563** |

Due to the limited number of **Chlorophyll *a* measurement** (n = 9 per treatment), and the large variability between replicates for each treatment, the significance was low between all treatments. A non-parametric test was chosen as normal distribution of the data could not be assumed with the given dataset.

| **Treatment** | **W** | **p value** |
| --- | --- | --- |
| Control - Reduced Light | 4 | 0.5 |
| Control - Reduced Blue Light | 7 | 0.1914 |
| Control - Reduced Red Light | 5 | 0.5 |
| Reduced Light - Reduced Red Light | 2 | 0.1914 |
| Reduced Light - Reduced Blue Light | 6 | 0.3313 |
| Reduced Blue Light - Reduced Red Light | 6 | 0.3313 |

The changes in maximum **Net Photosynthetic rate (*NP_max_*)** were statistically significant between Control and Reduced Light, and Control and Reduced Blue Light. In all instances, n ≥ 3, as measurements were made per triplicate culture:

| **Treatment** | **t** | **Df** | **P value** |
| --- | --- | --- | --- |
| Control - Reduced Light | -3.5153 | 3.4559 | **0.03115** |
| Control - Reduced Blue Light | 3.6704 | 3.9673 | **0.02169** |
| Control - Reduced Red Light | -2.1026 | 3.9243 | 0.1047 |
| Reduced Light - Reduced Red Light | -2.0624 | 3.1803 | 0.126 |
| Reduced Light - Reduced Blue Light | -0.80626 | 3.2748 | 0.4745 |
| Reduced Blue Light - Reduced Red Light | 1.7321 | 3.9905 | 0.1585 |

Change in **cell diameter** were not statistically significant between any treatments.

| **Treatment** | **t** | **Df** | **P value** |
| --- | --- | --- | --- |
| Control - Reduced Light | -2.0196 | 3.3556 | 0.127 |
| Control - Reduced Blue Light | -0.541 | 3.8161 | 0.6185 |
| Control - Reduced Red Light | -2.2177 | 3.874 | 0.09304 |
| Reduced Light - Reduced Red Light | -0.17321 | 3.7092 | 0.8715 |
| Reduced Light - Reduced Blue Light | -2.5174 | 2.9412 | 0.08805 |
| Reduced Blue Light - Reduced Red Light | -2.8823 | 3.4845 | 0.05298 |

Table S1: Parameters from Eilers photoinhibition model:

| condition | *NP_max_*  (pmol O_2_ cell^-1^ hr^-1^) | *I_opt_*  (μmol m^−2^ s^−1^) | *a* | c | AIC | Quasi R^2^ |
| --- | --- | --- | --- | --- | --- | --- |
| Control | 1.015±0.23 | 454.558±56 | 0.026±0.0 | -0.231±0.1 | -337.1±4.8 | 0.956±0.0 |
| Reduced Light Intensity | 1.872±0.35 | 535.939±33 | 0.034±0.01 | -0.04±0.02 | 323.13±10.8 | 0.938±0.04 |
| Reduced Blue Light | 1.684±0.21 | 392.474±46 | 0.030±0.01 | -0.092±0.1 | -329.2±1.01 | 0.936±0.02 |
| Reduced Red Light | 1.393±0.20 | 389.381±55 | 0.025±0.0 | -0.142±0.13 | -323.3±8.3 | 0.879±0.04 |

Table S2: Data from the PI curve (Fig 1 and 2 e):

| Treatment | Light Intensity | Avg. NP Rate | Std. Dev of NP Rate |
| --- | --- | --- | --- |
|  | (μmol m^−2^ s^−1^) | (pmol O_2_ cell^-1^ hr^-1^) | (pmol O_2_ cell^-1^ hr^-1^) |
| Control | 0 | -0.22846 | 0.061834 |
| Control | 80 | 0.568828 | 0.103903 |
| Control | 150 | 0.961866 | 0.223653 |
| Control | 300 | 1.044397 | 0.272855 |
| Control | 550 | 0.975797 | 0.324034 |
| Control | 700 | 0.965072 | 0.185083 |
| Control | 850 | 0.904326 | 0.23575 |
| Control | 1000 | 0.978412 | 0.210778 |
| Control | 1250 | 0.836401 | 0.248078 |
| Control | 1500 | 0.798747 | 0.294154 |
| Control | 1950 | 0.843825 | 0.237678 |
| Reduced Light Intensity | 0 | -0.04722 | 0.076002 |
| Reduced Light Intensity | 80 | 1.404439 | 0.173621 |
| Reduced Light Intensity | 150 | 1.775343 | 0.50206 |
| Reduced Light Intensity | 300 | 1.997224 | 0.522181 |
| Reduced Light Intensity | 550 | 1.888675 | 0.429 |
| Reduced Light Intensity | 700 | 1.965884 | 0.446301 |
| Reduced Light Intensity | 850 | 1.667009 | 0.315059 |
| Reduced Light Intensity | 1000 | 1.662418 | 0.188769 |
| Reduced Light Intensity | 1250 | 1.728221 | 0.386499 |
| Reduced Light Intensity | 1500 | 1.776848 | 0.42963 |
| Reduced Light Intensity | 1950 | 1.861153 | 0.109517 |
| Reduced Blue Light | 0 | -0.05957 | 0.127551 |
| Reduced Blue Light | 50 | 0.583288 | 0.238828 |
| Reduced Blue Light | 80 | 1.301844 | 0.301393 |
| Reduced Blue Light | 150 | 1.571493 | 0.184045 |
| Reduced Blue Light | 300 | 1.610033 | 0.256561 |
| Reduced Blue Light | 550 | 1.576778 | 0.235131 |
| Reduced Blue Light | 700 | 1.572528 | 0.263295 |
| Reduced Blue Light | 850 | 1.558254 | 0.283819 |
| Reduced Blue Light | 1000 | 1.433333 | 0.302507 |
| Reduced Blue Light | 1250 | 1.36794 | 0.312555 |
| Reduced Blue Light | 1500 | 1.293872 | 0.317807 |
| Reduced Blue Light | 1950 | 1.117281 | 0.278207 |
| Reduced Red Light | 0 | -0.11317 | 0.152115 |
| Reduced Red Light | 50 | 0.380031 | 0.170253 |
| Reduced Red Light | 80 | 1.031117 | 0.237014 |
| Reduced Red Light | 150 | 1.356102 | 0.222027 |
| Reduced Red Light | 300 | 1.39712 | 0.252123 |
| Reduced Red Light | 550 | 1.377061 | 0.106393 |
| Reduced Red Light | 700 | 1.133731 | 0.405758 |
| Reduced Red Light | 850 | 1.221949 | 0.207435 |
| Reduced Red Light | 1000 | 1.179595 | 0.094025 |
| Reduced Red Light | 1250 | 1.073863 | 0.115321 |
| Reduced Red Light | 1500 | 0.980788 | 0.231353 |
| Reduced Red Light | 1950 | 1.056733 | 0.177277 |









Fig S3: Lopadolith – Murolith intermediates (obtained from either blue-light or red-light limited treatments)


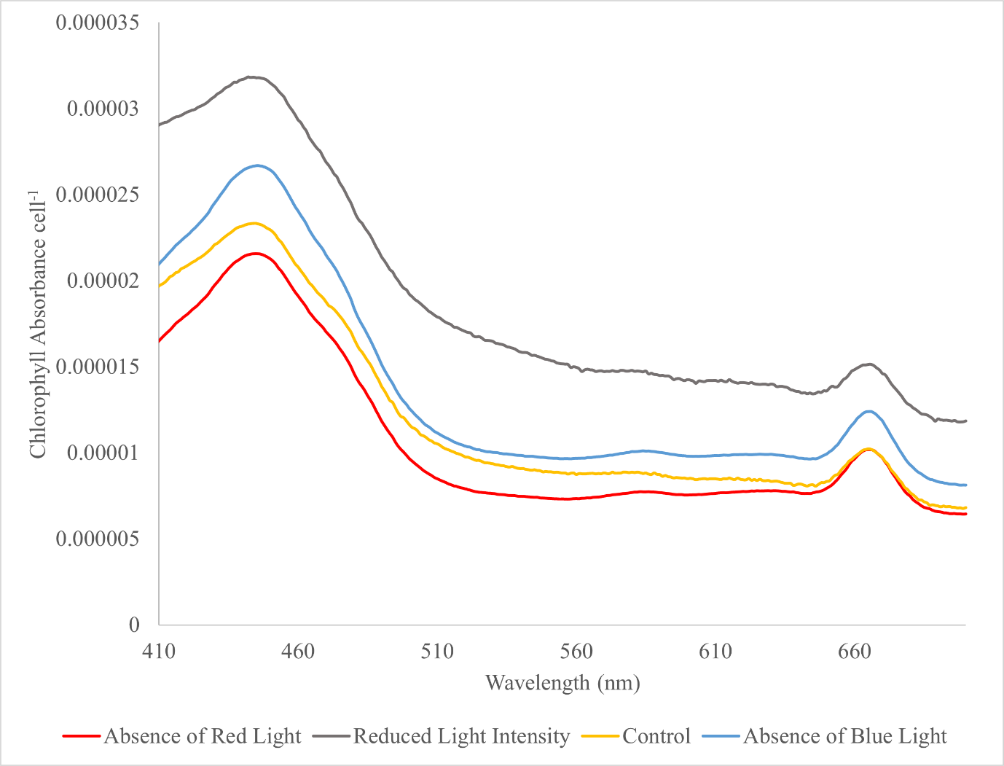


Fig S4: Chlorophyll absorbance scan


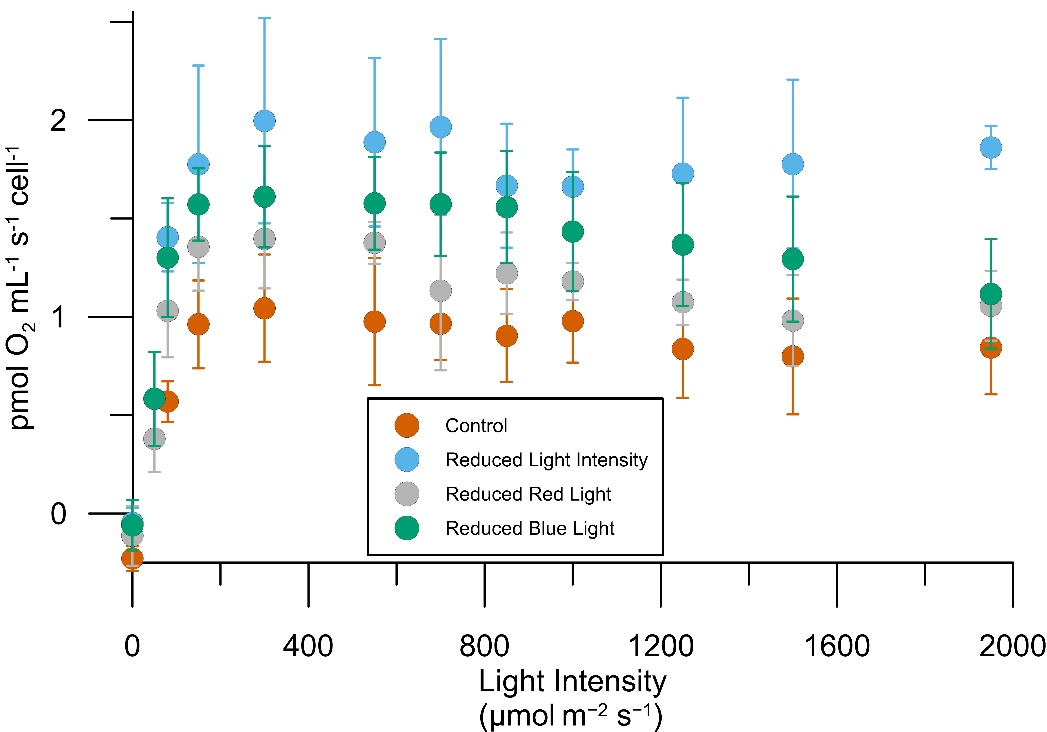


Figure S5: Full PI curves for S. apsteinii under different light quality treatments.

**References**

Andersen, R. (2005) *Algal culturing techniques*. Elsevier.

Bujang, M. A. and Adnan, T. H. (2016) Requirements for Minimum Sample Size for Sensitivity and Specificity Analysis. *J Clin Diagn Res*, **10**, YE01.

Kline, P. (1986) *A handbook of test construction: Introduction to psychometric design.* Methuen, New York.

Marra, J. (1978) Effect of short-term variations in light intensity on photosynthesis of a marine phytoplankter: A laboratory simulation study. *Mar Biol*, **46**, 191–202.

Ritchie, R. J. (2006) Consistent sets of spectrophotometric chlorophyll equations for acetone, methanol and ethanol solvents. *Photosynth Res*, **89**, 27–41.

Warren, C. R. (2008) Rapid Measurement of Chlorophylls with a Microplate Reader. *J Plant Nutr*, **31**, 1321–1332.
